# Study of Clinical and Biological Characteristics of Moroccan Covid-19 Patients With and Without Olfactory and/or Gustatory Dysfunction

**DOI:** 10.3389/fphys.2020.595005

**Published:** 2020-11-19

**Authors:** Hasnae Benkirane, Jaafar Heikel, Fatima Zahra Laamiri, Amina Bouziani, Houria Lahmam, Ayoub Al-Jawaldeh, Noureddine El Haloui, Khalid Ennibi, Naim Akhtar-Khan, El Mostafa El Fahime, Majdouline Obtel, Amina Barkat, Hassan Aguenaou

**Affiliations:** ^1^CNESTEN, Joint Research Unit in Nutrition and Food, Regional Designated Center of Nutrition (AFRA/IAEA), Ibn Tofaïl University, Kenitra, Morocco; ^2^Mohammed VI University of Health Sciences, Casablanca, Morocco; ^3^Laboratory of Health Sciences and Technology, Higher Institute of Health Sciences of settat, Hassan First University of Settat, Settat, Morocco; ^4^Nutrition, Department of NonCommunicable Diseases and Mental Health World Health Organization (WHO),Regional Office for the Eastern Mediterranean (EMRO), Abdul Razzak Al-Sanhouri, Cairo, Egypt; ^5^Center of Virology, Infectious and Tropical Diseases, Mohammed V Military Hospital, Rabat, Morocco; ^6^Inserm Research Center, U1231 INSERM/UB/AgroSup, Team-Physiology of Nutrition & Toxicology, Faculty of Life Sciences, University Bourgogne Franche-Comté (UBFC), Dijon, France; ^7^Functional Genomic Platform, UATRS-CNRST, Rabat, Morocco; ^8^Laboratory of Community Health, Preventive Medicine and Hygiene, Laboratory of Epidemiology and Clinical Research, Department of Public Heath, Faculty of Medicine and Pharmacy, Mohammed V University, Rabat, Morocco; ^9^Health and Nutrition Research Team of the Mother-Child Couple, Faculty of Medicine, Mohammed 5th University, Rabat, Morocco

**Keywords:** ageusia, anosmia, biological, epidemiological, demographic characteristics, Moroccan patients, COVID-19

## Abstract

**Background:**

The epidemic of severe acute respiratory syndrome coronavirus-2 (SARS-CoV-2), causing coronavirus disease 2019 (COVID-19), presents a significant and urgent threat to global health. This alarming viral infection, declared as pandemic by the WHO in February 2020, has resulted millions of infected patients and thousands of deaths around the world. In Morocco, despite the efforts made by the authorities, the SARS-CoV-2 continues to spread and constitutes a burden of morbidity and mortality. The objective of this study is to describe clinical characteristics of COVID-19 Moroccan patients and to establish the relationship between specific clinical symptoms, namely ageusia and/or anosmia, with these characteristics.

**Methods:**

We performed a descriptive, non-interventional cross-sectional study analyzing data from 108 patients admitted to the VINCI clinic, Casablanca (Morocco). The database includes 39 parameters including epidemiological characteristics, anthropometric measurements and biological analyzes.

**Results:**

The average of age of the patients was 43.80 ± 15.75 years with a sex ratio of 1:1. The mean body mass index of the patients was 25.54 ± 4.63 Kg/m^2^. The majority of patients had, at least, one comorbidity and among 75% symptomatic patients, about 50% had, at least, three symptoms namely, fever (40.7%), cough (39.8%), myalgia (28.7%), and anosmia and/or ageusia (20.4%). From biological analyzes, we noticed lymphopenia and an elevated protein C reactive and lactate dehydrogenases levels in 24.1, 36.1, and 35.2% of patients, respectively. A disturbance in liver function markers was observed in 15.7% of cases. For the other hemostasis parameters, high levels of prothrombin and platelets were reported in 14.6 and 14.8% of patients, respectively. Comparisons related to the presence of anosmia and/or ageusia did not show any difference for demographic and anthropometric characteristics, while a possibility of a significant difference was revealed for certain biological parameters, particularly the levels of lymphocytes, D-dimer and troponin.

**Conclusion:**

This study provides significant findings that will be used not only to supplement previous studies carried out in Morocco in order to resume the epidemiological situation in comparison with other countries, but also to improve the quality of the diagnosis of COVID-19 patients by identifying all the symptoms of the disease and better understanding its clinical outcomes.

## Introduction

The world is currently experiencing an alarming epidemic called coronavirus disease 2019 (COVID-19), caused by an infectious new viral strain belonging to the coronavirus family, i.e., severe acute respiratory syndrome coronavirus-2 (SARS-CoV-2), first detected in the Wuhan district of eastern China (Lu and Stratton, 2020). Infection of this virus quickly reached all corners of the world and saturated even the most resilient health systems (Organisation mondiale de la santé (OMS), 2020). Globally, 20% of infected subjects developed a severe or critical form of the disease, with a fatality rate currently above 3%, with higher rates in the older people and in those with chronic diseases (World Health Organization, 2020a).

Common signs of SARS-CoV-2 infection are respiratory symptoms, fever, cough, myalgia, shortness of breath, sore throat, and dyspnea. In more severe cases, the infection can cause pneumonia, severe acute respiratory syndrome and kidney failure (Hussain et al., 2020). However, some people, although infected, have only very mild symptoms or remain asymptomatic (Hussain et al., 2020).

Recent reports have also demonstrated the appearance of a new symptom in COVID-19 patients, i.e., sudden loss of the sense of smell and/or taste with the absence of the common clinical viral symptoms (Lechien et al., 2020a). It is a specific sign of COVID-19 infection that was recently added, by the WHO, to the list of other symptoms (World Health Organization, 2020a). The presence of anosmia and ageusia may appear in the early stages of COVID-19 (Zhou et al., 2020a) and represent an important diagnostic tool (Lee et al., 2020). Furthermore, the loss of taste and smell varies well according to sex and age. Interestingly, these sensorial alterations were generally more prevalent in women than men and young patients compared to adults (Giacomelli et al., 2020; Lechien et al., 2020b).

In parallel to these apparent symptoms, other immunological, biochemical and biological markers have been highlighted in COVID-19 patients (Yang et al., 2020c), they generally cover inflammatory and obesity indicators (Yang et al., 2020c; Arthur et al., 2020). Therefore, old age, chronic metabolic diseases and male sex make a favorable environment for SARS-CoV-2 infection that, in turn, can trigger acute and fatal hyperinflammation, called “cytokine storm” (Xu et al., 2020b). Several hypotheses have been proposed, suggesting that the smell and/or taste dysfunction could be due to inflammation of the nasal or oral neurological tissues. However, the physiopathological mechanism of this phenomenon remains unknown (Baig et al., 2020).

Additionally, obesity which is a major risk factor of several chronic diseases, including cardiovascular complications, diabetes, cancer, kidney dysfunction, etc., is also associated with a higher risk of respiratory tract infections, and hence the virus installation. Actually, an increase in adiposity has been shown to modify the integrity of the respiratory epithelium, which could lead to dysfunction of the respiratory tract (Honce and Schultz-Cherry, 2019) with an inflammatory response resulting in immunosuppression that could promote viral infections (Khan, 2006). In term of oro-sensory perception, olfaction or taste disorders can lead to weight gain or to an aggravation of the infection by distorting the feeling of food satiation (Tomassini et al., 2017). Furthermore, body mass index (BMI) affects olfactory function (Skrandies and Zschieschang, 2015). It has been suggested that obese subjects are at high risk of SARS-CoV-2 infection because they already have a low olfactory and gustatory capacity due to obesity which will mask the decrease in taste and odor induced by SARS-CoV-2 infection (Khan et al., 2020).

Most importantly, it has been increasingly evidenced that COVID-19 patients, before showing any clinical sign related to the viral infection, exhibit a loss of chemosensory perception of smell and taste. As mentioned above, some obese subjects suffer from the loss of gustatory perception, which may be explained by the association of obesity with “low grade” inflammation, marked with high concentrations of pro-inflammatory cytokines. COVID-19 is also marked with a “inflammatory cytokine storm.” So, we decided to categorize our COVID-19 patient population as with or without this oro-naso-sensory perception to better shed light at this aspect in relation with inflammation (Khan et al., 2020).

Besides, in Morocco, the data available on coronavirus mainly focused on generalities (description of the virus, its mode of spread and diagnosis, the adopted treatments, the number of cases, etc.) (Bourhanbour and Bakkouri, 2020; Traore et al., 2020; Zoukal et al., 2020). However, further studies testing more characteristics of COVID 19 patients remain limited. Therefore, the present study aims to describe anthropometric and clinical characteristics of Moroccan patients with COVID-19 and to define differences for each parameter according to the olfactory or gustatory dysfunction. This study will also allow us to specify the part of the population most at risk (males or females, young or old population, etc.) to assess the condition of the patients studied and to study the disease’s severity in order to guide treatment and therapeutic management of patients.

## Materials and Methods

### Study Design and Recruitment Site

This is a descriptive, non-interventional cross-sectional study. It was carried out among COVID-19 patients admitted to the VINCI clinic in Casablanca (Morocco) from 20 March to 04 June 2020.

The study, including patient monitoring and all performed analyzes, was carried out in partnership with the VINCI clinic in Casablanca, Mother and Child Health & Nutrition Research team, Faculty of Medicine and Pharmacy, Mohammed V University, Rabat, Ministry of Health as well as National Center for Scientific and Technical Research, Rabat (Morocco).

A trained medical staff of the VINCI clinic realized data collection, using a validated questionnaire by all parties involved in this study and presented as separated sections. Thus, the identity of each patient and its contact details were recorded. Likewise, measurements of anthropometric and biological parameters were carried out.

In addition, data on olfactory or gustatory dysfunction (taste and/or smell) were also collected from questions reported in a separate section and which are scored based on questionnaires already used by researchers (Mattos et al., 2019; Lechien et al., 2020a).

### Description of the Participants

The data-set covers patients admitted for COVID-19 at the VINCI Clinic in Casablanca. Indeed, only information on adult patients (men and women) confirmed by a positive COVID-19 (using PCR test and CT chest) and aged 18 years or older was noted. Thus, a total of 108 patients were enrolled in this study. This represents the total number of patients who, were hospitalized at the Vinci clinic at the time of writing this article. With the lifting of confinement, the cases’ number is currently increasing. The Vinci clinic is again involved in hospitalizations.

### Details of Data Collection and Measurements

Among patients meeting the inclusion criteria, the following steps, measures and interventions have been performed by the clinical staff in-charge:

#### Infection Confirmation

Infection confirmation was enrolled based on PCR test and radiological examination.

##### PCR’s details

PCR was carried out at Pasteur’s institute in Casablanca. Indeed, extraction of viral RNA from nasopharyngeal and oropharyngeal swabs was adopted according to the “Berlin protocol” which was developed and made available worldwide in mid-January 2020 by Professor Christian Drosten, Director of the Institute of Virology at the Charité Hospital in Berlin. This test targets the SARS-CoV-2 E and RdRp gene (Corman et al., 2020).

##### Radiological examination

This examination was realized using a United UCT 528 multi-barrette scanner with use of the CORADS score and determination, by specific software, of the percentage of the reached territory.

#### Demographic Data

For each participant, data was collected on age, sex and alcohol or tobacco use. Studied patients were classified into different age groups, i.e., 18–34, 35–44, 45–54, 55–64, and ≥65 years.

#### Anthropometric Measurements

The anthropometric parameters were measured following the WHO measurement standards (World Health Organization, 1995). These parameters included body weight and height. The BMI was calculated as the body weight in kilogram divided by the height squared in meter. According to obtained BMI values, the participants were classified into different groups, based on the WHO reference values (World Health Organization, 1995), as follows: underweight, BMI < 18.5 Kg/m^2^; normal, 18.5 ≤ BMI < 25 Kg/m^2^; overweight, 25 ≤ BMI < 30 Kg/m^2^; obesity class 1, 30 ≤ BMI < 35 Kg/m^2^; obesity class 2, 35 ≤ BMI < 40 Kg/m^2^, and obesity class 3: BMI ≥ 40 Kg/m^2^.

#### Clinical Survey

During the consultation, a clinical survey was prospectively realized for each patient. Data on common symptoms associated with COVID-19 infection principally fever, cough, myalgia, headache, asthenia, pharyngitis, digestive disorders, breathing difficulties and anosmia and/or ageusia were noted. Also, the presence of any other conditions and specifically severe non-communicable diseases, such as diabetes, cardiovascular disease, dyslipidemia, high blood pressure, etc., was recorded.

#### Olfactory and Gustatory Survey

Using a validated questionnaire, the anosmia and/or ageusia was/were evaluated in a specific section. This part was attributed to define the olfactory and/or gustatory dysfunction.

#### Biological Analysis

In the Institute Pasteur Laboratory in Casablanca, a number of biological parameters were analyzed in blood samples. On the first day of consultation and after 5 days of hospitalization, a set of biological parameter such as glycemia, hemoglobin (Hb), white blood cells (WBC), lymphocyte, prothrombin time (PT), reactive protein C (CRP), ferritin, D-dimers, aspartate aminotransferase (AST), alanine aminotransferase (ALT), gamma-glutamyl transpeptidase (GGT), troponin, creatine phosphokinase (CPK), lactate dehydrogenase (LDH) were measured for each recruited patient.

The biological analysis of these parameters was made on the basis of paraclinical examinations recommended by the national scientific commission of COVID-19. In addition to the etiological diagnosis and epidemiological surveillance, these laboratory examinations are used to monitor patients according to their cases and their comorbidities, to guide treatment and therapeutic management, and also to avoid a double bacterial infection.

Therewith, for each studied parameter, a defined diagnostic technique was used:

• **Hemogram (Hb, WBC, lymphocyte)**: Sample: whole blood EDTA- SYSMEX flow cytometry- Reference values: adult annals clinical biology 2014-Pediatrics RFL 2009;
• **Prothrombin:** Sample: citrated plasma-SIEMENS Reagent (Human Thromboplastin) Sysmex CA 620 Automate
• **CRP:** ROCHE immunoturbidimetric technique;
• **Ferritin:** ROCHE Electrochemiluminescence technique;
• **D-Dimers**: ELFA bioMérieux automated technique;
• **ASAT, ALAT, and GGT transaminases**: IFCC technique 37°C ROCHE;
• **Troponin:** Electrochemiluminescence technique on COBAS ROCHE;
• **C.P.K and LDH**: Technique UV 37 ROCHE.

### Statistical Analysis

Statistical analysis was performed using SPSS software-version 23.0 (IBM SPSS Statistics 23.0.0.0, New York, NY, United States). Baseline characteristics and clinical features of all patients with SARS-CoV-2 infection were described, as numbers and percentages by adding confidence interval estimates for better precision. Biological parameters were also analyzed and presented as mean ± standard deviation or as medians (quartiles), and each variable was categorized into different groups (Low, normal, and high), according to the biological variations, as follows:

– Hb (Man) categorized into 2 groups: <13.4 and >13.4 g/dl;
– Hb (Woman) categorized into 2 groups: <11.5 and >13.4 g/dl;
– WBC categorized into 3 groups: <4550/mm^3^, 4550–11000 mm^3^, and 30–155 mg/l;
– CRP categorized into 3 groups: <5, 5–30, and 30–155 mg/l;
– CPK categorized into 2 groups: <190 and >190 UI/l;
– LDH categorized into 2 groups: <225 and >225 UI/l;
– Procalcitonin categorized into 2 groups: >0,5 and >0,5 ng/ml;
– AST categorized into 2 groups: <40 and >40 UI/l;
– ALT categorized into 2 groups: <41 and >41 UI/l;
– Prothrombin time categorized into 3 groups: <70, 70–130 and >130%.

The normality of the distribution was evaluated using the Kolmogorov-Smirnov (KS) test.

Between the group of patients with olfactory (or gustatory) dysfunction and those without such dysfunction, comparisons have been made according to demographic, anthropometric and biological data employing different statistical tests depending on the studied variable. The comparison of quantitative variables with normal distribution was carried out by the student *t*-test and when the parametric hypotheses were not satisfactory, the Mann-Whitney test was performed. Association between qualitative variables was made using the Chi-square test or Fisher’s exact test. A value of *p* < 0.05 was considered for all statistical analyzes.

**NB:** Variables with missing data < 1% (GGT gamma-glutamyltranspeptidase, procalcitonin, and troponinemia) or <5% (BMI: Body mass index) were processed after exclusion of missing data. While for CPK and D-dimer, the missing data is greater than 10%, therefore statistical comparison tests were applied on valid data.

### Ethics

This study is a part of an overall project on the COVID-19 pandemic which has obtained the approval from the Ethical Committee and Biomedical Research (CEBR) of the College of Medicine and Pharmacy of Mohammed V University (Rabat, Morocco). The Committee approved the inclusion of the patients’ data, provided by the VINCI clinic, in order to analyze all the data and to make the different possible correlations (Approval number: 17/20, delivered on 12/06/2020). Written informed consent was obtained from each participating patient or from their family member in the case of severe conditions.

## Results

### General Characteristics of the Study Population

A total of 108 patients with laboratory-confirmed COVID-19 were included in this study. The general characteristics of the population are shown in Table 1. The age of our patients ranged from 18 to 82 years with an average of 43.80 ± 15.75 years. Almost one third of the patients (27.77%) were over 55 years old and 32.4% were young patients under 35 years old. The distribution by sex was statistically similar. The mean BMI of the patients was 25.54 ± 4.63 years with extremes of 18.90 and 42.90 Kg/m^2^. Almost half of the patients (42.59%) were either overweight (28.8%; 95% CI: 20.2–37.5) or obese (15.4%; 95% CI: 8.7–22.1). The majority of patients neither consumed tobacco, nor alcohol. Analysis of the comorbidity profile revealed that 28.7% had, at least, one comorbidity (95% CI: 20.4–37). Diabetes and arterial hypertension were the most common comorbidities.

**TABLE 1:** Characteristics of COVID-19 patients.

Characteristics	*n* = 108 (%)	95% confidence interval (CI)
**Age groups (years)**		
[18–35]	35 (32.4)	24.1–40.7
[35–45]	17 (15.7)	9.3–23.1
[45–55]	26 (24.1)	15.7–32.4
[55–65]	20 (18.5)	11.1–25.9
≥65	10 (9.3)	3.7–14.8
**Sex**		
Male	54 (50)	40.7–59.3
Female	54 (50)	40.7–59.3
**BMI categories (*n* = 104)**		
Normal weight	58 (55.8)	46.2–66.3
Overweight	30 (28.8)	20.2–37.5
Obesity class 1	9 (8.7)	3.8–14.4
Obesity class 2	6 (5.8)	1.9–10.6
Obesity class 3	1 (1)	0–2.9
**Tabaco consumption**		
No	97 (89.8)	83.3–95.4
Yes	11 (10.2)	4.6–16.7
**Alcohol consumption**		
No	89 (82.4)	75–88.9
Yes	19 (17.6)	11.1–25
**Diabetes**		
No	95 (88)	81.5–93.5
Yes	13 (12)	6.5–18.5
**Arterial hypertension**		
No	92 (85.2)	77.8–91.7
Yes	16 (14.8)	8.3–22.2
**Dyslipidemia**		
No	103 (95.4)	91.7–99.1
Yes	5 (4.6)	0.9–8.3
**Chronic respiratory disease**		
No	101 (93.5)	88.9–98.1
Yes	7 (6.5)	1.9–11.1
**Cardiovascular disease**		
No	106 (98.1)	95.4–100
Yes	2 (1.9)	0–4.6

*Values are expressed in count and percentage. BMI (body mass index): Underweight: BMI < 18.5 Kg/m^2^; Normal weight: 18.5 ≤ BMI < 25 Kg/m^2^; Overweight: 25 ≤ BMI < 30 Kg/m^2^; Obesity class 1: 30 ≤ BMI < 35 Kg/m^2^; Obesity class 2: 35 ≤ IMC < 40 Kg/m^2^; Obesity class 3: IMC ≥ à 40 Kg/m^2^.*

### Clinical Characteristics of the Patients

Analysis of Table 2, representing the distribution of patients on admission according to clinical signs, showed that 25% (95% CI: 15.7–33.3) had no clinical signs. Infection was symptomatic in three quarters of the study population (75%; 95% CI: 66.7–84.3).

**TABLE 2:** Clinical symptoms of COVID-19 patients.

Clinical signs	*n* = 108 (%)	95% confidence interval (CI)
**Fever and chills**		
No	64 (59.3)	50–68. 5
Yes	44 (40.7)	31.5–50
**Cough/dyspnea**		
No	65 (60.2)	50.9–68.5
Yes	43 (39.8)	31.5–49.1
**Myalgia**		
No	77 (71.3)	63–79.6
Yes	31 (28.7)	20.4–37
**Ageusia/anosmia**		
No	86 (79.6)	72.2–87
Yes	22 (20.4)	13–27.8
**Headache**		
No	91 (84.3)	77.8–90.7
Yes	17 (15.7)	9.3–22.2
**Asthenia/tiredness**		
No	81 (75)	66.7–82.4
Yes	27 (25)	17.6–33.3
**Pharyngitis**		
No	95 (88)	81.5–94.4
Yes	13 (12)	5.6–18.5
**Digestive disorders**		
No	93 (86.1)	79.6–91.7
Yes	15 (13.9)	8.3–20.4
**Fever or chills**		
No	104 (96.3)	92.6–99.1
Yes	4 (3.7)	0.9–7.4
**Polypnoea**		
No	104 (96.3)	92.6–99.1
Yes	4 (3.7)	0.9–7.4
**Oxygen desaturation**		
No	97 (89.8)	84.3–94.4
Yes	11 (10.2)	5.6–15.7
**High blood pressure**		
No	94 (87)	80.6–93.5
Yes	14 (13)	6.5–19.4

*Values are expressed in count and (percentage).*

Among the 81 symptomatic patients, we found that more than half (59.25%) had more than three clinical signs with a preponderance of the following signs: fever, cough, myalgia, asthenia, ageusia and anosmia. The distribution of patients according clinical signs and comorbidity was statistically similar (*p* = 0.177). In fact, the number of symptomatic patients was statistically higher as compared to asymptomatic patients both in the group with comorbidity (83.9 vs. 16.9%) and in the group without comorbidity (71.4 vs. 28.6%).

A minority of the patients suffered from cardiovascular disorders other than high blood pressure or chronic respiratory conditions, while nephropathy was almost absent in all of the patients.

### Distribution of Covid-19 Patients by Age Groups According to General Characteristics and Clinical Symptoms

The distribution of Covid-19 patients according to their general parameters, comorbidities and clinical signs in relation to age groups, is described in Table 3.

**TABLE 3:** Distribution of Covid-19 patients by age groups according to general characteristics and clinical symptoms.

Characteristics	Age groups (years) *N* = 108					*P*
	[18–35[	[35–45[	[45–55[	[55–65[	≥65	
**Sex**						0.686*
Male	19 (35.2)	9 (16.7)	12 (22.2)	11 (20.4)	3 (5.6)	
Female	16 (29.6)	8 (14.8)	14 (25.9)	9 (16.7)	7 (13)	
**BMI categories (*n* = 104)**						0.540**
Normal weight	23 (39.7)	9 (15.5)	13 (22.4)	11 (19)	2 (3.4)	
Overweight	8 (26.7)	6 (20)	6 (20)	5 (16.7)	5 (16.7)	
Obesity	4 (25)	2 (12.5)	4 (25)	4 (25)	2 (12.5)	
Tabaco consumption (yes)	3 (27.3)	5 (45.5)	2 (18.2)	1 (9.1)	0 (0)	0.127**
Alcohol consumption (yes)	6 (31.6)	3 (15.8)	2 (10.5)	8 (42.1)	0 (0)	0.041**
Diabetes (yes)	0 (0)	1 (7.7)	2 (15.4)	4 (30.8)	6 (46.6)	<0.001**
Arterial hypertension (yes)	0 (0)	0 (0)	3 (18.8)	4 (43.8)	6 (37.5)	<0.001**
Dyslipidemia (yes)	0 (0)	1 (20)	0 (0)	2 (40)	2 (40)	0.017**
Chronic respiratory disease (yes)	4 (57.1)	2 (28.6)	0 (0)	1 (14.3)	0 (0)	0.305**
Cardiovascular disease (yes)	0 (0)	0 (0)	0 (0)	2 (100)	0 (0)	0.094**
Fever and chills (yes)	11 (25.0)	8 (18.2)	10 (22.7)	11 (25.0)	4 (9.1)	0.510**
Cough/dyspnea (yes)	11 (25.6)	8 (18.6)	8 (18.6)	9 (20.9)	7 (16.3)	0.184**
Myalgia (yes)	9 (29.0)	3 (9.7)	7 (22.6)	7 (22.6)	5 (16.1)	0.443**
Ageusia/anosmia (yes)	10 (45.5)	1 (4.5)	5 (22.7)	4 (18.2)	2 (9.1)	0.455**
Headache (yes)	3 (17.6)	2 (11.8)	4 (23.5)	6 (35.3)	2 (11.8)	0.309**
Asthenia/tiredness (yes)	9 (33.3)	3 (11.1)	3 (11.1)	7 (25.9)	5 (18.5)	0.117**
Pharyngitis (yes)	4 (30.8)	2 (15.4)	2 (15.4)	4 (30.8)	1 (7.7)	0.800**
Digestive disorders (yes)	8 (53.3)	1 (6.7)	4 (26.7)	2 (13.3)	0 (0)	0.364**
Fever or chills (yes)	0 (0)	1 (25.0)	1 (25.0)	2 (50.0)	0 (0)	0.261**
Polypnoea (yes)	0 (0)	1 (25.0)	0 (0)	2 (50.0)	1 (25.0)	0.082**
Oxygen desaturation	1 (9.1)	1 (9.1)	1 (9.1)	5 (45.5)	3 (27.3)	0.011**
High blood pressure	1 (7.1)	1 (7.1)	2 (14.3)	6 (42.9)	4 (28.6)	0.003**

Values are expressed as number and percent. * Pearson chi-square test. **: exact test of Fisher. A value of p < 0.05 is considered significant.

Overall, the statistical analysis revealed a statistically significant difference between the age groups concerning the following variables: Alcohol (*p* = 0.041), diabetes (*p* < 0.001), dyslipidemia (*p* = 0.017), oxygen desaturation (*p* = 0.011) and high blood pressure (*p* = 0.003).

The distribution of patients for these main parameters was marked by high numbers, particularly for the group of participants aged between 55 and 65 years and for those aged ≥65 years.

### Biological Characteristics of the Study Population

Table 4 summarizes the main laboratory parameters of patients on admission. Analysis of the results showed hyperglycemia in 25.2% (95% CI: 17.8–34.6). WBC count were low (<4050/mm^3^) in 12% of cases (95% CI: 6.5–18.5), normal (4050–11000/mm^3^) in 81.5% of cases (95% CI: 74.1–88) and high (>11000/mm^3^) in only 6.5% (95% CI: 1.9–12) of cases. The lymphocyte count was below 1241/mm^3^ in 24.1% (95% CI: 16.7–32.4), normal in 75% (95% CI: 66.7–82.4) and above 3919/mm^3^ in only 0.9% (95% CI: 0–2.87) of cases. The prevalence of anemia was 11.1% (95% CI: 2.8–13.1) in men and 14.8% in women (95% CI: 5.6–25.9). Exploration of hemostasis parameters showed a disturbance of PT whose levels exceeded 100% in 14.6% (95% CI: 7.8–22.3), as well as platelets whose number was less than 161 × 10^3^/mm3 in 14.8% (95% CI: 8.3–21.3) of cases. Our results also revealed an increase in inflammation markers, in particular CRP, which exceeded 5 mg/l, in 36.1% of cases (95% CI: 26.9–45.4), ferritin which was above 400 ng/ml in 13.9% of cases (95% CI: 7.4–20.4) and D-dimers whose increase (>500 ng/ml) was noticed in 13.2% (95% CI: 5.9–22.1) of patients. A disturbance of biological markers of hepatic function was also marked by an increase in aspartate aminotransferase (AST > 40 UI/l), alanine aminotransferase (ALT > 41 UI/l) and gamma-glutamyl transpeptidase (GGT > 60 UI/l) in 15.7% (95% CI: 9.3–23.1), 19.4% (95% CI: 12–26.9) and in 18.9% (95% CI: 11.3–26.4) of patients, respectively. In addition, our results reported a slight alteration in blood troponin and creatinine phosphokinase (CPK) which were, respectively, higher than 14 ng/l in 7.5% (95% CI: 2.8–13.1) and 190 IU/l in 7.8% (95% CI: 2.2–13.3). Furthermore, our results revealed an increase in lactate dehydrogenase (LDH) and procalcitonin (PCT) which exceeded, respectively, 225 UI/l in 35.2% (95% CI: 26.9–44.4) and 0.5 mg/ml in 2.8% (95% CI: 0–6.5) of patients.

**TABLE 4:** Biological characteristics of COVID-19 patients.

Biological parameters	Number of patient *n*	Result
**Hb (g/dl)**^*a*^	108	14.02 ± 1.44
Hb < 13.4 g/dl^*b*^ (men)		6 (11.1)
Hb < 13.5 g/dl^*b*^ (women)		8 (14.8)
**Platelet count** (×10^3^/mm^3^)^*a*^	108	235.34 ± 77.15
Platelet count (<161 × 103/mm^3^)^*b*^		16 (14.8)
**WBC count/ (mm^3)c^**	108	6390 [4895–7830]
WBC < 4050/ (mm^3^)^*b*^		13 (12)
**Lymphocyte count (/mm^3^)**^*a*^	108	1787.91 ± 762.67
Lymphocyte < 1241/ (mm^3^)^*b*^		26 (24.1)
**Blood glucose (g/l)**^*c*^	107	1.10 [0.94–1.26]
Blood glucose > 1,26 (g/l)^*b*^		27 (25.2)
**CRP (mg/l)**^*c*^	108	2.25 [0.96–10.17]
CRP > 5 (mg/l)^*b*^		39 (36.1)
**Ferritin (ng/ml)**^*c*^	108	136.70 [52.75–253.40]
Férritin > 400 (ng/ml)^*b*^		15 (13.9)
**CPK (IU/L)**^*c*^	90	101 [64–148]
CPK > 190 (IU/l)^*b*^		7 (7.8)
**LDH (IU/L)**^*c*^	108	202.50 [170.5–263.25]
LDH > 225 (UI/l)^*b*^		38 (35.2)
**Troponin (ng/l)**^*c*^	107	4.30 [3.7–6.4]
Troponin > 14 (ng/l)^*b*^		8 (7.5)
**D-dimer (ng/ml)**^*c*^	67	203.50 [117–389.75]
D-dimer > 500 (ng/ml)^*b*^		9 (13.2)
**PCT (ng/ml)**^*c*^	107	0.05 [0.05–0.05]
PCT > 0.5 (mg/ml)^*b*^		3 (2.8)
**AST (IU/L)**^*c*^	108	23.50 [19.25–31.75]
AST > 40 (UI/l)^*b*^		17 (15,7)
**ALT (IU/L)**^*c*^	108	22.50 [15–38.75]
AST > 41 (UI/l)^*b*^		21 (19.4)
**GGT (IU/L)**^*c*^	107	21 [15–49.25]
GGT > 60 (UI/l)^*b*^		20 (18.9)
**PT (%)**^*a*^	106	91.49 ± 12.11
PT > 100 (%)^*b*^		15 (14.6)

*Values are expressed as mean ± standard deviation (a), as number and percent (b) or median and quartile (c). Hb, hemoglobin; WBC, White blood cell count; CRP, protein C reactive; CPK, creatinine phosphokinase; LDH, lactate dehydrogenases; PCT, procalcitonin; AST, aspartate aminotransferase; ALT, alanine aminotransferase; GGT, gamma-glutamyltranspeptidase; PT, Prothrombin time. Reference intervals for normal subject: Hb: 13.4–17 g/dl (men), 11.5–15.5 g/dl (women); Platelet count: 161–398 × 10^3^/mm^3^; WBC: 4050–11000/mm^3^; Lymphocyte count: 1241–3919/mm^3^; Blood glucose: 0.74–1.26 g/l; CRP: <5 mg/l; Ferritin: 30–400 ng/ml; CPK: <190 UI/l; LDH: <225 UI/l; Troponin: <14 ng/l; D-dimer: <500 ng/ml PCT: <0,5 ng/ml; AST: <40 UI/l; ALT: <401 UI/l; GGT: <60 UI/l; PT: 70–130%*

### Comparison of COVID-19 Patients With Alterations in Oro-Naso-Sensory Parameters

In order to explore the demographic and anthropometric profile in the group with olfactory and/or taste dysfunction, we compared these parameters (Table 5). The comparison of the biological parameters made it possible to highlight a statistically significant difference which concerned only the number of lymphocytes, the levels of troponin and D-dimer in the blood. Indeed, we observed in the group with taste and or olfactory dysfunction compared to the group without any alteration in this function, a significantly high increase in the percentage of patients with lymphopenia (29.1% vs. 4.5%; *p* = 0.019), D-dimer >500 ng/ml (77.8 vs. 22.2% *p* = 0.002) and troponinemia >14 ng/l (18.2% vs. 4.1%; *p* = 0.032).

**TABLE 5:** Olfactory and gustatory dysfunctions in COVID-19 patients.

Characteristics	olfactory or gustatory dysfunction		*p*
	No *n* = 86 (%)	Yes *n* = 22 (%)	
**Age (years)a**	44.34 ± 15.62	41.68 ± 16.44	0.483^α^
**Age group^*s*^**			0.455^γ^
[18–35]	25 (71.4)	10 (28.6)	
[35–45]	16 (94.1)	1 (5.9)	
[45–55]	21 (80.8)	5 (19.2)	
[55–65]	16 (80.0)	4 (20)	
≥65	8 (80)	2 (20)	
BMI (Kg/m^2^)^*a*^	25.59 ± 4.78	25.26 ± 4.20	0.771^α^
BMI *categories* (*n* = 104)^*b*^			0.942^γ^
Normal weight	47 (81)	11 (19)	
Overweight	23 (76.7)	7 (23.3)	
Obesity	13 (81.3)	3 (18.8)	
Sex			0.633^μ^
Male	44 (81.5)	10 (18.5)	
Female	42 (77.8)	12 (22.2)	
Hb (g/dl)^*a*^	13.91 ± 1.42	14.43 ± 1.49	0.135^α^
WBC count (/mm^3^)^*c*^	6520 [4905–8055]	5820 [4307.5–7395]	0.282^ß^
Platelet count (/mm^3^)^*a*^	238.37 ± 76	223.5 ± 82.24	0.422^α^
Blood glucose (g/l)^*c*^	1.09 [0.94–1.24]	1.1 [0.93–1.30]	0.761^ß^
C-reactive protein (mg/l)	2.5 [0.98–14.57]	1.79 [0.90–5.92]	0.387^ß^
Ferritin (ng/ml)^*c*^	135.7 [51.25–259.27]	140.6 [57.52–226.1]	0.749^ß^
CPK (IU/L)^*c*^	98 [62.50–147.50]	108 [70.50–162]	0.371^ß^
LDH (IU/L)^*c*^	204 [162.75–265.75]	190.5 [178.50-.]	0.900^ß^
Troponin (>14 ng/l)^*b*^	4 (4.7)	4 (18.2)	0.032^μ^
D-dimer (>500 ng/ml)^*b*^	7 (13.2)	9 (40.9)	0.008^μ^
AST (IU/L)^*c*^	23 [19.75–34]	24 [17.75–28.50]	0.921^ß^
ALT (IU/L)^*c*^	22.50 [15–39.25]	22 [16.75–37.25]	0.593^ß^
GGT (IU/L)^*c*^	21 [15–54.50]	21 [15–38]	0.583^ß^
PT (%)^*a*^	90.69 ± 12.38	94.54 ± 10.74	0.185^α^

*Values are expressed as mean ± standard deviation (a), as number and percent (b) or median and quartile (c). α: Student’s t test; ß: Mann-Whitney test; Ɣ: exact test of Fisher; μ: Pearson chi-square test. A value of p < 0.05 is considered significant. Hb, hemoglobin; WBC, White blood cell count; CRP, protein C reactive; CPK, creatinine phosphokinase; LDH, lactate dehydrogenases; PCT, procalcitonin; AST, aspartate aminotransferase; ALT, alanine aminotransferase; GGT, gamma-glutamyltranspeptidase; PT, Prothrombin time. Reference intervals for normal subject: Hb: 13.4–17 g/dl (men), 11.5–15.5 g/dl (women); Platelet count: 161–398 × 10^3^/mm^3^; WBC: 4050–11000/mm^3^; Lymphocyte count: 1241–3919/mm^3^; Blood glucose: 0.74–1.26 g/l; CRP: <5 mg/l; Ferritin: 30–400 ng/ml; CPK: <190 UI/l; LDH: <225 UI/l; Troponin: <14 ng/l; D-dimer: <500 ng/ml; AST: <40 UI/l; ALT: <401 UI/l; GGT: <60 UI/l; PT: 70–130%*

## Discussion

Our study aims to describe the demographic, anthropometric, clinical and biological characteristics of Moroccan COVID-19 patients. It presents a detailed analysis which complements previous Moroccan researches by analyzing different characteristics cited and possible correlations. In particular the differences of each parameter according to the olfactory or taste dysfunction were examined. In general, the results of our study show some points of similarity and others of differences with the previous studies for the different parameters. However, the present research work reports original results in relation to the symptoms like anosmia or ageusia wherein we did not observe any difference according to demographic and anthropometric characteristics. A possibility of a significant difference was noticed, however, for certain biological parameters, particularly the levels of lymphocytes, of the D-dimer and of the troponin.

We recruited 108 adult patients with a mean age of 43.80 ± 15.75 years and a sex ratio of 1:1. There was no difference in the proportion of men and women, which was inconsistent with the results of a study that have been conducted by Guan et al. (2020) who observed that men were more likely to be infected than women. The same result has been demonstrated by a Danish team (Kragholm et al., 2020).

In terms of the analysis of BMI values, we observed that almost half of the patients (44.3%) were overweight or obese (28.8 and 15.4% respectively). A similar obesity prevalence (48.3%) among COVID patients has been published by Finer et al. (2020), while, other researchers noted a high prevalence of obesity among patients with SARS-CoV-2 infection (Arthur et al., 2020; Peng et al., 2020). Furthermore, our results suggest that overweight and obesity could be risk factors for severe infection with COVID-19 in line with emerging data published in other clinical studies (Hamer et al., 2020; Sattar et al., 2020). Indeed, potential mechanisms have been linked to immune hyperresponsiveness, altered metabolic responses and pulmonary dysfunction, including a reduction in forced expiratory volume and a forced biological capacity, to the problem of overweight or obesity (Khan et al., 2020; Sattar et al., 2020). Also, lipid peroxidation is a key factor giving rise to reactive lipid aldehydes which will affect the prognosis of patients infected with SARS-CoV-2 (Demetrios et al., 2020). Physiologically, it has been shown that angiotensin converting enzyme 2 (ACE2) is the assumed receptor for the entry of SARS-CoV-2 in host cells. Given the expression level of this receptor is very high in the tissues, the possibility of risk in obese patients infected by this virus increases greatly (Zhou et al., 2020c).

In relation to comorbidities, this survey pointed out that almost a third of the population had, at least, one comorbidity with a slight dominance for diabetes and hypertension compared to the other studied comorbidities (dyslipidemia, chronic respiratory disease and cardiovascular disease). Similar results were found in a cohort of 85 patients in Wuhan, China, with first-degree diabetes followed by high blood pressure in patients presenting 68% for comorbidities (Du et al., 2020).

For non-communicable diseases, lifestyle risk factors have been consistently associated with morbidity, mortality, and loss of disease-free life years (Colpani et al., 2018; Nyberg et al., 2020; Schlesinger et al., 2020). For example, physical inactivity and smoking appear to be independently associated with a higher risk of community-acquired pneumonia and pneumonia mortality (Baik et al., 2000; Wang et al., 2014; Paulsen et al., 2017). However, the evidence from alcohol consumption and diet on the risk of respiratory infection is less clear (Paulsen et al., 2017; Hamer et al., 2019). Fortunately, most of the participants in this study were non-smokers (about 89.8%) and were not alcohol dependent (82.4%).

On the other hand, data on the characteristics of clinical complications revealed that a quarter of patients did not present any symptoms. In the symptomatic population, the predominant clinical signs were fever (40.7%), cough (39.8%), myalgia (28.7%), fatigue (25%), and anosmia and/or ageusia (20.4%). Nevertheless, high blood pressure, cardiovascular disorders and respiratory conditions were present in a limited number of our patients and nephropathy was almost absent. Such results have been reported by numerous researches studying COVID-19 patients (Wang et al., 2020; Yu et al., 2020; Zhang et al., 2020b). However, other studies observed an association between the SARS-CoV-2 infection and the elevated risk of developing diseases such as chronic kidney disease, heart disorders, diabetes, etc. (Arentz et al., 2020; Gorbalenya et al., 2020).

Regarding biological parameters, our results showed that hyperleukocytosis and lymphopenia, which represent a key indicator of infection, were noticed in some patients. In this context, numerous studies have reported that more than 80% of infected patients with SARS-CoV-2 presented these symptoms particularly lymphopenia (Mostafa et al., 2020; Yang et al., 2020c). Indeed, many researchers have revealed that in patients who died from lymphopenia, a trace of severe SARS-CoV-2 infection was confirmed. This can be explained by the death of endothelial cells due to endothelial dysfunction in certain chronic diseases which then causes excessive leakage of WBC and a disruption of the blood tissue barrier that may reflect the lymphocytes decrease found in patients with severe COVID infection (Bermejo-Martin et al., 2020). More studies have also reported that a significant increase in WBC presents a clinical worsening sign that has been shown to be significantly elevated in dead subjects (Henry et al., 2020). However, another study has found normal WBC values with lymphocyte decrease in diagnosed COVID-19 patients (Golnaz et al., 2020).

Concerning inflammatory markers, our results also detected a particular increase in CRP in 36.1% of patients with a general median value of 2.25 mg/l. Such an increase might be due to viral inflammation (Sproston and Ashworth, 2018). In our study, CRP reflected the COVID-19 pathogenesis presenting an immune response to this viral infection (Mostafa et al., 2020). In clinical laboratories, the test of this marker is currently widely used for the assessment of SARS-CoV-2 infection (Zhang et al., 2020b). For D-dimer, ferritin and LDH, as other parameters of inflammation, we also noted an increase in 13.2, 13.9, and 35.2% of cases, respectively. This finding is in line with results of previous researches which showed that blood levels of D-dimers, presenting also a sign of coagulation, were higher in severe SARS-CoV-2 infected cases (Wang et al., 2020; Yang et al., 2020b), while a study by Sun Ziyong of Huazhong University of Science and Technology in Wuhan, China, has shown that D-dimer is linked to a poor prognosis for COVID-19 patients (Tang et al., 2020b). Similarly, elevated ferritin levels could be interpreted as a sign of infection with SARS-CoV-2. In agreement with this observation, Zhou and his collaborators have also noted high ferritin levels in 200 adult patients (Zhou et al., 2020b). Indeed (Wenzhong and Hualan, 2020), based on an *in silico* analysis (not yet validated by peers) of the SARS-CoV-2 genome, reported the sequences encoding non-structural proteins that attack hemoglobin, in particular one of the beta chains, from which they would extract the iron atom, leading to ferritin increase in blood of COVID-19 patients.

Besides D-dimer, prothrombin and platelets are other important coagulation and thrombotic indicators commonly used in clinical laboratories for the early diagnosis of infection. Several reports have pointed out that high level of prothrombin is generally linked with the severity of the infection, of which hypercoagulation is the main result of this increase (Ling et al., 2020; Zhou et al., 2020a). Interestingly, the number of platelets has been shown to be negatively correlated with the risk of mortality in SARS-CoV-2 infected patients (Tang et al., 2020a). However, the precise mechanism by which the SARS-CoV-2 virus acts on platelet function remains unclear (Manne et al., 2020).

Additionally, the present study demonstrated a disruption of biological markers of liver function with an increase in AST, ALT and GGT transaminases, in accordance with previous studies that have shown an increase in transaminases in 25 to 35% of COVID-19 patients (Fan et al., 2020; Xu et al., 2020a; Zhang et al., 2020a; Zhou et al., 2020a).

As for the comparison of patients with and without anosmia and/or ageusia according to their demographic and anthropometric, we found no statistically significant difference between the two groups. Nevertheless, the comparison of the biological parameters revealed a significant difference between two groups with remarkable lymphopenia, a high level of D-dimer and troponemia in patients with anosmia and/or ageusia. This is in the opposite direction with the study of Hornuss et al. (2020) who reported similar clinical and laboratory test outcomes in patients with and without anosmia or hyposmia. Likewise, another study COVID-19 patients showed no difference in lymphocyte and D-dimer levels between those with peripheral nervous system disorders with loss of taste and smell as the main symptom and those without these disorders and, therefore, without anosmia or ageusia (Ling et al., 2020). These noticeable alterations in biological parameters including inflammatory (D-dimer) and immune (lymphocytes) indicators in subjects suffering from anosmia and/or ageusia can be attributed to the severity of the clinical symptoms which strongly activate the immune system, leading to a severe inflammatory (Trotier et al., 2007; Khan et al., 2020; Ling et al., 2020).

There are several limitations, linked to the current study. First, the processed data relate to a single hospital center, which does not allow conclusions to be drawn on all Moroccan patients. Secondly, with regard to the symptom of dysfunction of the senses, details on the type (olfactory or gustatory or both) and on the specificity of the total or partial loss have not been made. Thirdly, no specific test on the loss of taste and smell was performed, and these observations are derived from self-reported questionnaire. Fourth, although PCR tests can usually determine whether a person is currently infected with the Sars-Cov-2 virus, they are still not 100% accurate. Indeed, false negatives can occur with a frequency of around 30% because, within 2 weeks of exposure. Thus, this test does not determine whether people who have been exposed to SARS-CoV-2 will develop Covid-19 or not (Longokolo et al., 2020) and it is not recommended by the WHO for clinical use (Haute Autorité de Santé (HAS), 2020).

## Conclusion

This study is the first of its kind to be conducted among Moroccan patients with SARS-CoV-2 infection. It provides important information on the demographic, anthropometric, clinical and biological characteristics of these patients. The majority of cases present the common coronavirus symptoms with remarkable disturbances in inflammatory and other biological markers as an immune response to defend against the viral infection. Thus, this is an essential work that presents the part of the Moroccan Health Department’s efforts to improve the quality of the diagnosis of COVID-19 based on the identification of all the disease symptoms. It also presents an important baseline study for future studies focusing on the possible correlations between the SARS-CoV-2 epidemic and its various symptoms, especially the olfactory and gustatory dysfunctions, considered to be as an important sign for the diagnosis of patients, especially for asymptomatic cases.

## Data Availability Statement

The raw data supporting the conclusion of this article will be made available by the authors, without undue reservation after terminating its exploitation for future publications.

## Ethics Statement

The studies involving human participants were reviewed and approved by the Ethical Committee and Biomedical Research (CEBR) of the College of Medicine and Pharmacy of Mohammed V University (Rabat, Morocco) (Approval umber: 17/20, delivered on 12/06/2020). The patients/participants provided their written informed consent to participate in this study.

## Author Contributions

HB, HA, and NE designed, coordinated and drafted the manuscript for publication. HA had oversight responsibility over the project. JH, EE, and KE collected the data and provided laboratory analyzes. HB and HL wrote the manuscript. ABO translated the manuscript. ABA, FL, and MO analyzed the results and performed statistical analysis. HA and ABA reviewed the manuscript. AE performed the scientific review of the manuscript. NA-K contributed to the critical advice for editing the manuscript. All authors read and approved the final version of the manuscript.

## Conflict of Interest

The authors declare that the research was conducted in the absence of any commercial or financial relationships that could be construed as a potential conflict of interest.
